# Antifungal Agents in the 21st Century: Advances, Challenges, and Future Perspectives

**DOI:** 10.3390/idr17040091

**Published:** 2025-08-01

**Authors:** Francesco Branda, Nicola Petrosillo, Giancarlo Ceccarelli, Marta Giovanetti, Andrea De Vito, Giordano Madeddu, Fabio Scarpa, Massimo Ciccozzi

**Affiliations:** 1 Unit of Medical Statistics and Molecular Epidemiology, Università Campus Bio-Medico di Roma, 00128 Rome, Italy; m.ciccozzi@unicampus.it; 2 Infection Prevention & Control/Infectious Disease Service, Fondazione Policlinico Universitario Campus Bio-Medico, 00127 Rome, Italy; n.petrosillo@policlinicocampus.it; 3 Department of Public Health and Infectious Diseases, University of Rome Sapienza, 00185 Rome, Italy; giancarlo.ceccarelli@uniroma1.it; 4 Department of Science and Technologies for Sustainable Development and One Health, Università Campus Bio-Medico di Roma, 00128 Rome, Italy; giovanetti.marta@gmail.com; 5 Oswaldo Cruz Institute, Oswaldo Cruz Foundation, Rio de Janeiro 21040-900, Brazil; 6 Unit of Infectious Diseases, Department of Medicine, Surgery and Pharmacy, University of Sassari, 07100 Sassari, Italy; andreadevitoaho@gmail.com (A.D.V.); giordano@uniss.it (G.M.); 7 Department of Biomedical Sciences, University of Sassari, 07100 Sassari, Italy; fscarpa@uniss.it

**Keywords:** antifungal resistance, invasive fungal infections, antifungal therapy, fungal pathogens, therapeutic challenges, drug development, emerging fungal threats

## Abstract

Invasive fungal infections (IFIs) represent a growing global health threat, particularly for immunocompromised populations, with mortality exceeding 1.5 million deaths annually. Despite their clinical and economic burden—costing billions in healthcare expenditures—fungal infections remain underprioritized in public health agendas. This review examines the current landscape of antifungal therapy, focusing on advances, challenges, and future directions. Key drug classes (polyenes, azoles, echinocandins, and novel agents) are analyzed for their mechanisms of action, pharmacokinetics, and clinical applications, alongside emerging resistance patterns in pathogens like *Candida auris* and azole-resistant *Aspergillus fumigatus*. The rise of resistance, driven by agricultural fungicide use and nosocomial transmission, underscores the need for innovative antifungals, rapid diagnostics, and stewardship programs. Promising developments include next-generation echinocandins (e.g., rezafungin), triterpenoids (ibrexafungerp), and orotomides (olorofim), which target resistant strains and offer improved safety profiles. The review also highlights the critical role of “One Health” strategies to mitigate environmental and clinical resistance. Future success hinges on multidisciplinary collaboration, enhanced surveillance, and accelerated drug development to address unmet needs in antifungal therapy.

## 1. Introduction

Invasive fungal infections (IFIs) represent a major global health challenge, especially for immunocompromised individuals such as cancer patients, organ transplant recipients, and those with HIV/AIDS. Recent estimates suggest IFIs contribute to over 1.5 million deaths annually, underlining their significant impact on morbidity and mortality, particularly in vulnerable populations [1]. The increasing number of immunosuppressed patients, driven by advancements in medical treatments such as transplants and immunosuppressive therapies, has further contributed to the rise in IFI incidence [2]. The COVID-19 pandemic has further exacerbated this issue, increasing susceptibility to secondary fungal infections, including aspergillosis, candidemia, and mucormycosis, among critically ill patients [3].

In recognition of the growing threat posed by fungal pathogens, the World Health Organization (WHO) published its first-ever Fungal Priority Pathogens List(FPPL) in 2024 [4]. This landmark document classifies 19 fungal pathogens into three priority tiers—critical, high, and medium—based on factors such as antifungal resistance, mortality, and public health burden. However, despite their growing clinical relevance, fungal infections have long been overlooked in public health strategies and have received significantly less research funding compared to diseases like malaria and tuberculosis [5] with similar mortality rates. This stark disparity in funding is concerning, especially given the high mortality associated with IFIs, which are now considered among the leading global causes of death [6]. Specifically, the majority of fungal-related deaths are caused by three main genera: *Aspergillus*, *Candida*, and *Cryptococcus* [7]. Globally, *Aspergillus* accounts for approximately 1.8 million deaths, *Candida* for about 995,000, and *Cryptococcus* for around 147,000 annually. Overall, IFIs are estimated to cause 3.8 million deaths annually, of which nearly 2.5 million result directly from the fungal infections. The economic burden is also substantial. In 2019 alone, fungal infections accounted for $7.5 billion in direct medical costs in the United States, with a total economic burden of over $48 billion when factoring in lost productivity and premature deaths [8]. Additionally, fungal infections lead to over 75,000 hospitalizations annually, with candidiasis and aspergillosis contributing to the highest costs [9].

Recent studies show a change in the distribution of *Candida* species, with an increase in non-albicans species and the growing prevalence of *Candida auris*. Since its first isolation in 2009, *C. auris* has rapidly gained prominence as an emerging pathogen, with clinical cases reported in numerous countries, including the United States, where more than 4000 new cases were reported in 2023, classifying it as an “urgent threat” to public health [10]. Its rapid spread has been associated with hospital outbreaks, especially in high-risk settings such as intensive care units, with mortality rates as high as 30% [11]. Resistance to antifungals is a major concern: about 90% of strains are resistant to fluconazole, 30% to amphotericin B, and 5% to echinocandins. In addition, cases of pan-antifungal resistance have been described. *C. auris* is able to survive for long periods of time on inanimate surfaces and can be difficult to identify correctly by conventional diagnostic methods, requiring advanced techniques such as PCR or MALDI-TOF mass spectrometry [12]. In Europe, hospital outbreaks have been reported in countries such as Italy, Spain, and the United Kingdom, with cases of prolonged colonization and persistent candidemia. The ability of *C. auris* to persist in hospital settings and colonize patients for prolonged periods makes control difficult, despite reinforced prevention measures [13].

These data show an increase from previous estimates. Emerging trends include changes in the distribution of *Candida* species, with an increase in non-albicans species and the emergence of *C. auris*, the increasing prevalence of azole resistance in *Aspergillus fumigatus*, often linked to the use of fungicides in agriculture, with studies indicating that at least 40% of azole-resistant *A. fumigatus* infections come from environmental exposures [14], and a change in the epidemiologic profile of patients with cryptococcosis. It is important to note that the increased burden of IFI is not solely attributable to the growing population of traditional immunocompromised patients. Factors such as critical illness, complications of viral infections, and potentially climate change [15] affecting the distribution of endemic fungi contribute to this complex epidemiology. This implies the need for multifactorial prevention and control strategies that go beyond immunosuppression management to include hospital infection control, post-viral management, and consideration of environmental factors.

This work aims to provide an up-to-date overview of antifungal agents in the context of the 21st century, exploring key developments and current challenges in this field. Throughout the manuscript, the term “invasive fungal infections (IFIs)” is used consistently to refer to deep or life-threatening mycoses affecting sterile body sites. The broader term “fungal infections” is used only when including both invasive and non-invasive forms. Specifically, Section 2 examines the different classes of antifungal drugs, outlining their distinguishing characteristics and mechanisms of action. The pharmacodynamics and pharmacokinetics of these agents are also explored, with a focus on their interactions with biological targets. Section 3 addresses the growing issue of antifungal resistance, examining its causes, epidemiology, and clinical implications. Section 4 reviews current therapeutic indications and recent guidelines, with an emphasis on innovative treatment strategies. Section 5 discusses the major challenges in antifungal research and the need for novel therapeutic solutions. Finally, Section 6 and Section 7 summarizes the key findings and proposes future directions for research and clinical practice in the management of fungal infections.

## 2. Mechanisms of Action and Pharmacodynamics

Understanding the molecular mechanisms by which antifungal agents exert their activity and the pharmacokinetic/pharmacodynamic (PK/PD) principles that govern their efficacy is critical for optimizing therapy and addressing the growing challenge of resistance.

### 2.1. Main Classes of Antifungals and Their Targets

Major classes of antifungals include polyenes, azoles, echinocandins, antimetabolites such as flucytosine, and allylamines such as terbinafine [16]. Polyenes, discovered in the 1950s, include Amphotericin B (AmB), Nystatin and Natamycin. AmB, available in conventional (deoxycholate, AmB-d) and lipid formulations (ABLC lipid complex, liposomal L-AmB), acts by binding to ergosterol, an essential component of the fungal cell membrane, causing it to destabilize and form pores. Lipid formulations of AmB have been developed to mitigate its toxicity, particularly the nephrotoxicity associated with the deoxycholate formulation. A meta-analysis showed that lipid formulations significantly reduced nephrotoxicity compared with conventional formulation [17].

Azoles, introduced since the 1960s, are subdivided into Imidazoles (e.g., Miconazole, Ketoconazole), used mainly topically or with limited systemic use, and Triazoles (e.g., Fluconazole, Itraconazole, Voriconazole, Posaconazole, Isavuconazole), widely used systemically. They inhibit ergosterol synthesis by blocking the cytochrome P450-dependent enzyme lanosterol 14α-demethylase, leading to the accumulation of toxic methylated sterols and depletion of ergosterol, altering membrane function. Azoles are generally fungistatic against yeasts and fungicidal against Aspergillus spp. but have a high risk of drug interactions and potential hepatotoxicity [18]. Isavuconazole, administered as the propharmaceutical isavuconazole sulfate, is a more recently introduced triazole approved for the treatment of invasive aspergillosis and mucormycosis.

Echinocandins, introduced in the early 2000s, include Caspofungin, Micafungin and Anidulafungin. They act by inhibiting the synthesis of β-(1,3)-D-glucan, an essential structural component of the fungal cell wall, through blocking the enzyme β-(1,3)-D-glucan synthase. They are fungicidal against *Candida* species and fungistatic against *Aspergillus* spp., generally well tolerated, with a more favorable drug interaction profile than azoles, although potential hepatotoxicity is reported. Echinocandins are considered the first-line therapy for invasive candidiasis in most current guidelines [19].

Flucytosine (5-FC), a pyrimidine analog, once captured by the fungal cell and converted to 5-fluorouracil, inhibits DNA and RNA synthesis. It is used almost exclusively in combination therapy, typically with AmB, to treat cryptococcal meningitis and some severe *Candida* infections, but its use is limited by potential myelotoxicity and the rapid development of resistance when used as monotherapy [20].

Allylamines, such as terbinafine, inhibit ergosterol synthesis by acting on the enzyme squalene epoxidase, leading to squalene accumulation and ergosterol depletion, altering fungal cell membrane function. Their use is primarily confined to the treatment of dermatophytoses, with a limited role in invasive fungal infections [21].

### 2.2. Principles of PK/PD Applied to Antifungals

The effectiveness of antifungal therapy depends not only on the choice of a drug that is active in vitro against the pathogen, but also on the ability to achieve and maintain adequate concentrations of the drug at the site of infection long enough to exert the desired effect. The study of the relationships between pharmacokinetics (PK, what the body does to the drug: absorption, distribution, metabolism, elimination) and pharmacodynamics (PD, what the drug does to the pathogen: inhibitory or cidal effect) is crucial for optimizing therapeutic regimens [22]. PK/PD studies aim to identify which pharmacokinetic parameters (typically measured in serum or plasma) best correlate with clinical or microbiological outcome, in relation to the pathogen’s in vitro susceptibility, usually expressed as Minimum Inhibitory Concentration (MIC), which represents the lowest drug concentration that inhibits visible fungal growth under standardized laboratory conditions [22].

Three main PK/PD indices are commonly used to describe the exposure-response relationship for antimicrobials [22]: the ratio of maximum concentration to MIC (Cmax/MIC), relevant for concentration-dependent drugs; the ratio of area under the concentration-time curve (AUC) to MIC (AUC/MIC), important for drugs whose efficacy depends on total exposure; and the time when the concentration exceeds the MIC (%T>MIC), significant for time-dependent drugs. Preclinical and clinical studies have identified the most relevant PK/PD indices for different classes of antifungals [23]. For azoles, the efficacy of triazoles is generally best correlated with the AUC/MIC ratio, and since only the free fraction is active, fAUC is often considered; for fluconazole in candidiasis a target fAUC/MIC > 25 is associated with favorable outcome [23]. Azole activity is time-dependent and concentration-independent once the MIC is exceeded, so cumulative exposure (AUC) is the key parameter [23]. Amphotericin B (AmB), belonging to the polyenes, shows concentration-dependent fungicidal activity, so the Cmax/MIC ratio is considered to be more predictive of its efficacy, and a Cmax/MIC > 40 has been associated with higher probability of therapeutic response with liposomal AmB [23]. In addition, AmB exhibits a significant post-antifungal effect (PAFE), i.e., persistent suppression of fungal growth after the concentration has fallen below the MIC, supporting less frequent administrations [24]. Echinocandins also show concentration-dependent activity against *Candida* spp. [24]; relevant indices are AUC/MIC and Cmax/MIC, and for *C. albicans* an fAUC/MIC ratio of about 20 (or 7 for less virulent species) is associated with efficacy [23]. For *Aspergillus*, activity is fungistatic and MEC (Minimum Effective Concentration) is often used instead of MIC [24]. Flucytosine (5-FC) shows efficacy correlated with AUC/MIC [24]. Knowledge of these PK/PD indices allows optimizing dosages and establishing clinical susceptibility breakpoints, i.e., MIC values that distinguish treatable from resistant strains [22]. Two pharmacokinetic aspects critical to antifungal efficacy are plasma protein binding and tissue distribution.

Only the free fraction of the drug, not bound to albumin, is pharmacologically active [25]; high protein binding may therefore limit its efficacy. Echinocandins show very high protein binding (>95–99%) [24]; many azoles also exceed 90%, while fluconazole and flucytosine have low binding (<15%) [24]. This explains why PK/PD indices are often expressed in terms of fAUC or fCmax [23]. Tissue distribution is another critical factor: many fungal infections involve barrier tissues (e.g., CNS, eye, bone, biofilms), and penetration depends on properties such as lipophilicity, protein binding, and molecular size [24,26]. Fluconazole, voriconazole, and flucytosine show good distribution, including in the CSF [24]; AmB accumulates in the liver, spleen, and lungs but has limited penetration into the CNS and other tissues [24]; echinocandins, because of their large size and strong protein binding, penetrate poorly into sites such as the CNS, eye, and urinary tract [24]; terbinafine concentrates in the skin and nails [23]. Incomplete knowledge about penetration to all sites of infection remains a clinical challenge [26].

Additional complexity arises from the high pharmacokinetic variability among patients, even at equivalent doses [23], particularly for voriconazole, posaconazole, and itraconazole. Causes include genetic polymorphisms (e.g., CYP2C19 for voriconazole) [24], drug interactions (CYP inducers/inhibitors) [23], and pathophysiologic factors such as age, liver/renal function, body weight, mucositis, or alterations in gastrointestinal absorption [24]. This variability makes it difficult to predict individual exposure, with risk of failure or toxicity [23]. Therapeutic drug monitoring (TDM), i.e., measurement of blood concentrations, is therefore recommended for some antifungals (voriconazole, posaconazole, itraconazole, and sometimes flucytosine) [23], with the goal of individualizing the dose to maximize efficacy and minimize toxicity. TDM is particularly useful in critically ill patients or those with known PK alterations, drug interactions, inadequate response, or suspected toxicity [23].

The link between mechanism of action, PK/PD parameters, and resistance is central: for example, azoles must bind to the heme of the enzyme Erg11/Cyp51A [25], and their efficacy depends on maintaining adequate concentrations (AUC/MIC) [23]. Mechanisms of resistance include mutations that alter the binding site or efflux pumps that reduce intracellular availability [26]. These aspects need to be integrated in the development of new drugs, ideally also active on mutated forms of Erg11 that are non-substrates of efflux pumps and have a favorable PK profile to achieve PK/PD targets even against less susceptible strains. A comparative summary of the main characteristics of the antifungal classes is given in Table 1.

**Figure 1:**
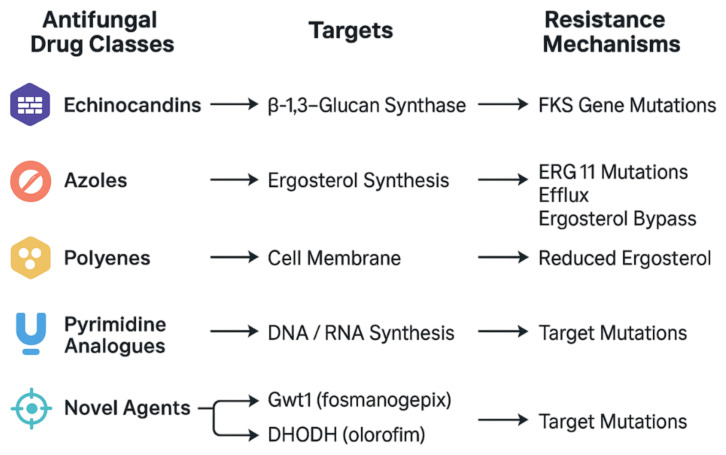
Schematic overview of major antifungal drug classes, their molecular targets, and associated resistance mechanisms. Echinocandins inhibit β-1,3-glucan synthase, with resistance typically arising from *FKS* gene mutations. Azoles block ergosterol biosynthesis via inhibition of lanosterol 14α-demethylase (*ERG11*), and resistance mechanisms include target mutations, efflux pump upregulation, and metabolic bypass. Polyenes bind ergosterol in the fungal cell membrane, leading to pore formation; resistance may result from reduced ergosterol content. Pyrimidine analogues interfere with nucleic acid synthesis, with resistance emerging through mutations in activating enzymes. Novel agents such as fosmanogepix (Gwt1 inhibitor) and olorofim (DHODH inhibitor) act on distinct biosynthetic targets, with resistance primarily due to target-site mutations.

A schematic summary of major antifungal drug classes, their targets, and associated resistance mechanisms is presented in Figure 1.

## 3. Antifungal Resistance

### Epidemiology and Global Distribution

Understanding the geographic spread and prevalence of antifungal resistance is critical to guide empirical treatment strategies, implement infection control measures, and inform health policies. The past few decades have witnessed the emergence and alarming spread of antifungal-resistant fungal pathogens, a growing threat to global public health that is acquiring a severity comparable to that of antibacterial pathogens. In particular, *Candida auris*, which has emerged almost simultaneously in different parts of the world since 2009 (first described isolation in Japan), has become a formidable nosocomial pathogen, reported in more than 60 countries [32]. This yeast is characterized by multi-resistance to multiple classes of antifungals, with almost universal resistance to fluconazole (>90%) and significant rates of resistance to amphotericin B (20-35%), as well as echinocandins (1–5%) [33]. Its transmission in hospitals is facilitated by its ability to spread rapidly, causing epidemic outbreaks that are difficult to control, especially in contaminated environments [33]. In addition, its diagnosis is complicated, as standard methods initially failed to correctly identify it, leading to underestimates of its prevalence [34]. Invasive *C. auris* infections, such as candidemia, are particularly severe, with mortality rates as high as 30-60% depending on the patient population [35]. The emergence of *C. auris* is also accelerated by the pressures on health systems caused by the COVID-19 pandemic, with the spread taking on a global scope thanks to globalization and international travel [35]. In parallel, another worrisome phenomenon concerns resistance to azoles of *Aspergillus fumigatus*, the main etiologic agent of invasive aspergillosis, which has seen increasing prevalence, especially in Europe [36]. Azole resistance in *A. fumigatus* is strongly linked to environmental exposure, as azole fungicides used in agriculture select resistant strains [32]. These strains, carrying specific mutations in the cyp51A gene, can easily infect immunocompromised patients, leading to difficult-to-treat infections. Azole resistance in *A. fumigatus* is particularly high in some European countries, such as the Netherlands, the United Kingdom, Denmark, and Spain, with resistance rates exceeding 10–20%, but also in Asia, the Middle East, and South America [28]. Infection with azole-resistant *A. fumigatus* is associated with very high mortality rates, with therapeutic failure as high as 90% [37]. *Cryptic aspergillosis* is also emerging as an increasingly relevant issue in both human and veterinary medicine [38]. Numerous cryptic species within the *Aspergillus fumigatus* complex have been identified as opportunistic pathogens. For example, *Aspergillus felis* has been recognized as a leading cause of invasive aspergillosis in cats [39]. Notably, azole resistance is significantly more common among these cryptic species. In a study conducted in Spain, Escribano et al. [40] reported an overall azole resistance prevalence of 7.4% in *A. fumigatus sensu lato* (63/847), but this rate rose sharply to 95% (18/19) among cryptic species, compared to just 5.5% (45/828) in *A. fumigatus sensu stricto*. This elevated resistance was primarily associated with the TR34/L98H mutation. In addition, other Candida species, such as *C. glabrata*, *C. parapsilosis*, *C. tropicalis*, and *C. krusei*, are showing worrisome resistance trends. *C. glabrata*, for example, is a common cause of candidemia and shows fluconazole resistance that can exceed 20% in some areas [41].

The availability of accurate, comparable, and timely antifungal surveillance data is critical to guide clinical and public health decisions. However, compared with bacterial resistance, antifungal surveillance has historically received less attention and resources, being fragmented, nonstandardized, and with limited geographic coverage, especially in low- and middle-income countries (LMICs) [33].

Launched by WHO in 2015, the GLASS [42] program aims to develop a standardized global system for collecting, analyzing, and sharing data on antimicrobial resistance (AMR) and antimicrobial consumption (AMC) [43]. Originally focused on bacteria, in 2019 GLASS introduced a protocol for surveillance of invasive fungal infections through the GLASS-FUNGI module, initially focused on *Candida* spp. isolated from blood [44]. GLASS takes a “One Health” approach that integrates data from human, animal, and environmental sources and provides visualization tools through an interactive online dashboard. However, until mid-2024, GLASS-FUNGI data are not yet publicly available [45]. Collaboration of GLASS with existing regional networks (e.g., EARS-Net, CAESAR) is active, but limitations remain, such as incomplete participation (with significant gaps in Africa and LMICs [46]), heterogeneity in data quality [47], and delays in fully integrating fungal infections. Organizations such as GAFFI have suggested the expansion of GLASS-FUNGI to include azole-resistant *Aspergillus fumigatus* [44]. The European Centre for Disease Prevention and Control (ECDC) coordinates antimicrobial resistance surveillance in the European Union/European Economic Area through EARS-Net [48]. EARS-Net historically focuses on eight bacterial pathogens isolated from invasive infections (blood and cerebrospinal fluid) [49], without including antifungal data related to *Candida* or *Aspergillus*. The information is accessible through the Surveillance Atlas of Infectious Diseases [50], which provides maps and graphs on bacterial resistance highlighting geographic gradients (low resistance in the North/West, high in the South/East) [51]. However, similar maps for antifungal resistance are not available. Thus, antifungal surveillance in Europe remains fragmented, based on multicenter studies or national networks, and not centralized [45,47]. In the United States, the Centers for Disease Control and Prevention (CDC) monitors antifungal resistance through the Emerging Infections Program (EIP) for *Candida* spp. [52] and the Antimicrobial Resistance Laboratory Network (AR Lab Network), which supports screening and susceptibility testing, particularly for *C. auris*. Mandatory national reporting for *C. auris* has been active since 2018 for clinical cases and since 2023 for colonization cases [53]. Data are made public and updated through geographic maps on the CDC website [53]. In addition, CDC-led studies have documented U.S. strains of *A. fumigatus* with environmental resistance mutations (TR34/L98H), albeit with lower prevalences than in Europe [54].The CDC actively collaborates with WHO and participates in GLASS. There are additional surveillance programs, including the SENTRY Antimicrobial Surveillance Program [43], multicenter epidemiological studies sponsored by entities such as the European Confederation of Medical Mycology (ECMM) [55], national networks (e.g., Kor-GLASS in South Korea [56]), regional initiatives (e.g., MAAP in Africa [48]), and commercial or academic databases (e.g., ATLAS from Pfizer [48] and ResistanceMap from CDDEP [57]).

**Figure 2:**
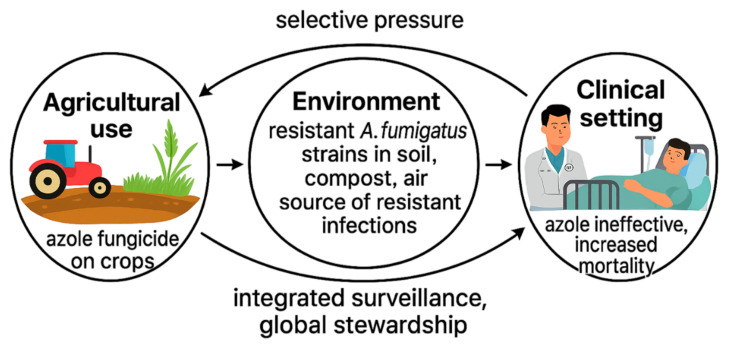
Schematic representation of the “One Health” model of antifungal resistance. The diagram illustrates the interplay between agricultural azole use, environmental selection of resistant *Aspergillus fumigatus* strains, and clinical outcomes. Agricultural fungicide application exerts selective pressure in the environment, leading to resistant strains in soil and compost, which can become airborne and cause invasive infections in humans.

In recent years, a crucial aspect that has emerged in the epidemiology of antifungal resistance, particularly for *Aspergillus fumigatus*, is the crucial role played by the extensive use of azole fungicides in agriculture and horticulture. There is now scientific consensus that the extensive use of fungicides belonging to the class of azoles (Demethylation Inhibitors, DMIs) in agriculture is a primary driver for the selection and spread of *A. fumigatus* strains resistant to clinical azoles [32]. The close structural similarity between agricultural and therapeutic azoles facilitates the emergence of cross-resistance mutations, particularly TR34/L98H and TR46/Y121F/T289A in the cyp51A gene, conferring resistance to both fungicides and medical azoles [58]. *A. fumigatus*, a ubiquitous saprophytic fungus found in soil, compost, on decaying vegetation, and in the air [55], is subjected to intense selective pressure in agricultural environments treated repeatedly with azoles, promoting the selection of resistant strains [58]. The spores of these strains are then dispersed into the environment, predominantly by air, increasing the risk of inhalation by immunocompromised or susceptible individuals. A particularly relevant clinical consequence is that “azole-naïve” patients-that is, without a history of prior exposure to therapeutic azoles-can develop invasive infections by *A. fumigatus* strains already resistant to first-line antifungals, such as voriconazole [32]. This phenomenon, explained by environmental inhalation of resistant spores, has been widely documented and associated with poor prognosis, with significantly high rates of therapeutic failure and mortality [37]. Originally observed in the Netherlands, the phenomenon of environmental resistance has gradually spread globally, having been documented on all continents except Antarctica [59]. The direct correlation between azole use in agriculture and clinical resistance underscores the importance of an integrated “One Health” approach to the management of antimicrobial resistance [60]. It is now clear that effective control strategies cannot be limited to the hospital setting (clinical antifungal stewardship), but must necessarily also include regulation of azole fungicide use in agriculture through Integrated Pest Management (IPM) practices and rational pesticide use policies [52]. The identification of the TR34/L98H and TR46 mutations as true “molecular signatures” of environmental-derived resistance in *A. fumigatus* [32] is a prime example of how anthropogenic activities in a sector, such as agriculture, can trigger a “domino effect” with direct and severe impacts on global public health. This causal chain-which links crop fields to hospital beds-highlights the need for coordinated, multisectoral regulatory, agricultural, and health interventions to break the cycle of emerging resistance. A schematic representation of this environmental-clinical connection within the “One Health” framework is provided in Figure 2.

Table 2 provides an indicative summary of observed resistance prevalences for some key pathogen-drug combinations in different geographic areas, although it is critical to emphasize the heterogeneity and inherent limitations of these data, which are based on fragmented sources and heterogeneous surveillance systems.

## 4. Clinical Applications

Management of IFIs, particularly invasive candidiasis (IC), requires a timely and targeted approach based on identification of the pathogen, assessment of its susceptibility to antifungals, and the patient’s clinical characteristics, such as site of infection, immune status, comorbidities, and concomitant therapies. Timely and accurate diagnosis plays a crucial role in informing antifungal selection. Early identification of the fungal pathogen and its susceptibility profile not only enables rapid initiation of appropriate therapy, but also reduces unnecessary exposure to broad-spectrum agents, limits toxicity, and helps contain the emergence of resistance. In the absence of microbiological confirmation, empirical choices are based on host risk factors and epidemiological data, but once diagnostic information becomes available, therapy can often be de-escalated or adjusted accordingly [64].

International guidelines, such as those published by the Infectious Diseases Society of America (IDSA), provide evidence-based recommendations for the management of major clinical syndromes [19]. For the treatment of invasive candidiasis, echinocandins (caspofungin, micafungin, anidulafungin) are recommended as empirical and targeted first-line therapy for most adult patients with candidaemia because of their fungicidal activity against most Candida species, including *C. glabrata* often resistant to fluconazole, and good tolerability profile [19]. Rezafungin, a new generation echinocandin with weekly administration, has recently been approved for the treatment of invasive candidiasis, demonstrating comparable efficacy to caspofungin and potential advantages in terms of less frequent administration [65]. As alternatives or de-escalation therapies, fluconazole remains a first-line option for clinically stable patients with no recent exposure to azoles and low risk of infection with resistant species, and is also the drug of choice for de-escalation once the isolate is identified as susceptible. Lipid formulations of amphotericin B are recommended for central nervous system infections, endocarditis, osteomyelitis, azole- and echinocandin-resistant species infections, or in cases of intolerance or failure of the other classes. Voriconazole is useful for some less common species or as de-escalation therapy for specific infections if the isolate is susceptible. Flucytosine is used almost exclusively in combination for serious infections such as meningitis, endocarditis, and urinary infections from resistant species. Management of *Candida auris* infections is complex because of frequent multiresistance. Echinocandins are generally considered the first-line therapy for invasive infections, but susceptibility testing is essential because of emerging resistance. Lipid formulations of amphotericin B represent an alternative. Combination therapies and new agents such as ibrexafungerp and fosmanogepix are under investigation. Ibrexafungerp, an oral triterpenoid, has shown in vitro activity against *C. auris*, including echinocandin-resistant isolates [66]. Fosmanogepix has shown clinical efficacy in patients with *C. auris* candidemia in phase 2 studies [67]. The duration of therapy for invasive candidiasis is generally 2 weeks after the first negative blood culture and resolution of symptoms and signs of infection, in the absence of metastatic foci. Removal of central venous catheters is strongly recommended.

Invasive aspergillosis (AI), caused primarily by *Aspergillus fumigatus*, poses a serious threat to immunocompromised patients, such as those who are neutropenic or undergoing transplantation. The first-line treatment for invasive pulmonary aspergillosis is voriconazole, whose efficacy is well documented, including in the treatment of disseminated central nervous system infections [68]. Isavuconazole is a first-line alternative, shown to be noninferior to voriconazole with a potentially better tolerability profile, presenting fewer drug-related adverse events. Lipid formulations of AmB (L-AmB) are also a first-line option, especially in settings with high prevalence of azole resistance or azole contraindications/intolerance. In cases of first-line failure, intolerance, or suspected/confirmed resistance, options include switching to another class of antifungals, use of posaconazole, or combination therapies, although evidence supporting combinations is limited and controversial [69]. Echinocandins as monotherapy are not recommended for the primary treatment of AI, but may have a role in combination or in selected cases. New agents such as olorofim and fosmanogepix are promising for resistant strains. For prophylaxis, posaconazole is recommended in high-risk patients, such as those with acute myeloid leukemia induction or allogeneic transplantation with GVHD. Voriconazole or micafungin may also be used in specific settings. Mucormycosis, an aggressive infection caused by fungi of the order Mucorales, often affects diabetic patients with ketoacidosis or immunocompromised patients. High-dose lipid formulations of AmB (≥5 mg/kg/day) are the therapy of choice, often in combination with aggressive surgical interventions [70]. Posaconazole and isavuconazole are viable alternatives that can be used as primary therapy in selected patients or as consolidation/step-down therapy after improvement with AmB [71]. Combination therapy may be considered in severe or refractory cases, but evidence is scarce [72]. Treatment of infections with *Fusarium* spp. or *Scedosporium/Lomentospora* spp. is particularly complex and requires susceptibility testing; voriconazole is active against *Scedosporium apiospermum*, but *Lomentospora prolificans* is typically multiresistant, often requiring combination therapies and new agents such as holorofim.

The management of opportunistic fungal infections, such as cryptococcosis and *Pneumocystis jirovecii* pneumonia (PCP), requires a targeted evidence-based treatment approach. *Cryptococcosis*, caused mainly by *Cryptococcus neoformans* and *C. gattii*, predominantly affects immunocompromised patients, particularly those with HIV infection. The recommended induction treatment involves the combination of amphotericin B (preferably in liposomal formulation) and flucytosine for 1–2 weeks, followed by a consolidation phase with fluconazole for at least 8 weeks and a maintenance phase until immune restoration. Recent studies have shown that a single high dose of liposomal amphotericin B combined with flucytosine and fluconazole is noninferior to the standard WHO regimen, with an improved safety profile [73]. Control of increased intracranial pressure is crucial for patient survival. PCP, caused by *Pneumocystis jirovecii*, represents another significant opportunistic infection in HIV-positive patients. First-line treatment consists of the administration of trimethoprim-sulfamethoxazole (TMP-SMX), which has demonstrated efficacy in both the treatment and prophylaxis of PCP. In cases of intolerance or therapeutic failure, alternatives include pentamidine, atovaquone, clindamycin combined with primaquine, and dapsone. Recent studies suggest that reduced doses of TMP-SMX can maintain therapeutic efficacy with fewer adverse events [74]. The use of echinocandins in combination with TMP-SMX is being studied for moderate to severe cases of PCP [75]. Primary and secondary prophylaxis with TMP-SMX remains the recommended strategy to prevent PCP in patients at risk [76].

Therapeutic strategies for IFIs include empirical, pre-emptive, and targeted approaches, each adapted to clinical suspicion, host factors, and diagnostic certainty. A summary of recommended first-line and alternative therapies for major invasive fungal infections is provided in Table 3. Empiric therapy is initiated in high-risk patients, such as those with persistent febrile neutropenia, before microbiologic confirmation of fungal infection. Echinocandins (e.g., caspofungin) are often preferred for suspected candidiasis, while voriconazole, isavuconazole, or liposomal amphotericin B are used for suspected aspergillosis. Studies have shown that empiric therapy can reduce the incidence of documented fungal infections in patients with persistent fever during neutropenia [77]. Pre-emptive therapy is based on the use of biomarkers such as galactomannan and β-D-glucan, together with imaging techniques, to identify IFI early and initiate treatment before microbiological confirmation. This strategy has shown comparable efficacy to empiric therapy, with the advantage of reducing unnecessary use of antifungals [78]. Once a fungal pathogen is identified, targeted therapy becomes the preferred approach, guided by antifungal susceptibility testing (AST), allowing for optimized drug selection, dose adjustments, and de-escalation to narrower-spectrum or less toxic agents [79]. Rapid diagnostic tools—including PCR, T2 magnetic resonance (T2MR), antigen-based assays, and MALDI-TOF MS—are crucial to accelerate pathogen identification and to transition from empirical/pre-emptive to directed therapy [80]. Finally, Antifungal Stewardship (AFS) programs play a pivotal role in promoting rational antifungal use, improving clinical outcomes, and minimizing resistance emergence [81].

## 5. Challenges and Future Prospects

Despite progress, the management of invasive fungal infections remains complex due to a number of challenges related to current therapies and pathogen biology. At the same time, research continues to explore new strategies and approaches to overcome these obstacles.

### 5.1. Limitations of Current Antifungal Therapy

Currently available classes of antifungal drugs have several inherent limitations that complicate their use and reduce their efficacy in certain clinical situations. Although newer drugs have improved the safety profile compared with conventional AmB, toxicity remains a concern. Nephrotoxicity is still the main dose-limiting side effect of Amphotericin B, even with lipid formulations, although the latter are associated with a lower incidence and severity than the deoxycholate formulation (D-AmB); infusion-related reactions are also common with D-AmB and, although reduced, possible with lipid formulations, while hepatotoxicity is less frequent but possible [61]. Azoles, which are generally better tolerated, are not without toxicity: hepatotoxicity (from mild transaminase elevations to severe hepatitis), visual disturbances and neurotoxicity with voriconazole, endocrine effects with ketoconazole, and risk of QTc interval prolongation with various triazoles require caution, especially in patients with cardiac risk factors; in addition, potential teratogenicity limits their use in pregnancy [82]. Echinocandins are generally very well tolerated due to their highly specific mechanism of action in the fungal cell wall [25,83], whereas flucytosine is associated with myelosuppression and potential gastrointestinal and hepatic toxicity. Management of these adverse effects is particularly complex in patients who are frail, have comorbidities, or are on prolonged treatment. Drug interactions pose an additional challenge, especially with azoles, which are substrates and potent inhibitors of cytochrome P450 isoenzymes (CYP3A4, CYP2C9, CYP2C19), with a high potential for clinically relevant interactions.

Inhibition of metabolism by other drugs (e.g., immunosuppressants, statins, anticoagulants, benzodiazepines) can cause toxicity, while enzyme induction by drugs such as rifampin, carbamazepine, or phenytoin can reduce the efficacy of azoles. Echinocandins have much less potential for metabolic interactions, given their CYP-independent metabolism, although some interactions are nonetheless documented, for example between caspofungin and rifampin or cyclosporine, and between micafungin and sirolimus. Other antifungals such as AmB and flucytosine have a low risk of CYP interactions, while terbinafine may be subject to interactions with enzyme inducers. Interactions complicate the management of patients with IFI, who are often immunocompromised and polytreated, making careful assessment of the drug profile essential. An additional difficulty arises from the incomplete spectrum of action and pharmacokinetic barriers: echinocandins are inactive against several relevant pathogens (Cryptococcus, Mucorales, Fusarium, Scedosporium), azoles have variable spectrum and exhibit intrinsic resistance (e.g., *C. krusei* to fluconazole), and not even AmB covers all pathogens (e.g., *A. terreus*, *C. lusitaniae*) [41].

Pharmacokinetics may also hinder clinical efficacy: tissue penetration is limited for some drugs (e.g., echinocandins and AmB in the CSF), while biofilms are an additional barrier. Oral bioavailability is another critical issue, with some drugs unavailable orally (echinocandins) or with variable absorption (itraconazole, posaconazole suspension) influenced by food and pH; new formulations (e.g., posaconazole tablets, isavuconazole) seek to address this limitation. In addition, very high protein binding may reduce the active drug quota, especially in contexts of hypoalbuminemia. Diagnostic difficulties further aggravate management: nonspecific clinical signs, slow and insensitive cultures, and antigenic and molecular tests with limitations in specificity, standardization, and availability make timely and accurate diagnosis difficult [33], contributing to therapeutic delays and inappropriate use of broad-spectrum antifungals.

IFIs also carry a significant economic impact [6]: prolonged hospital stays, intensive care use, expensive drugs (e.g., echinocandins, lipid AmB) [84], toxicity monitoring, interaction management, and resistance-related costs (longer therapies, second-line drugs, increased mortality) significantly impact healthcare systems; in 2018, hospitalizations for fungal infections accounted for approximately $6.7 billion in direct costs in the United States [6]. These combined limitations-toxicity, interactions, narrow spectrum, PK barriers, diagnostic difficulties, and high costs-create a complex picture in which treatment choices are often a trade-off between efficacy, safety, and feasibility, and where suboptimal use of antifungals can foster resistance [27], which in turn narrows therapeutic options, potentially forcing the use of more toxic or complex regimens. Breaking this cycle requires innovation on multiple fronts: safer drugs with fewer interactions, broad spectrum, better pharmacokinetics, faster diagnosis, and rigorous stewardship programs.

Current antifungal options also present significant limitations in vulnerable populations such as neonates and pregnant women. In neonatal candidemia, micafungin is often preferred over amphotericin B deoxycholate due to its favorable safety profile and lower nephrotoxicity, although dosing remains off-label in many regions. In pregnancy, the use of azoles is restricted due to their teratogenic potential, especially during the first trimester; in these cases, liposomal amphotericin B is generally considered the drug of choice for systemic fungal infections [19]. These considerations underscore the urgent need for antifungal agents with improved safety across special populations.

### 5.2. Innovations and Future Directions in Antifungal Therapy

The need to overcome the limitations of existing drugs (toxicity, spectrum of action, formulations) and, most importantly, to counter emerging antifungal resistance has stimulated the development of new molecules, as summarized in Table 4.

Rezafungin (CD101) [85], a next-generation echinocandin, was approved by the FDA in March 2023 and by the EMA in December 2023 for the treatment of invasive candidiasis in adults with limited therapeutic options. Structurally related to anidulafungin, it has modifications that increase its chemical and metabolic stability, giving it a much longer half-life (approximately 130–150 h), allowing weekly intravenous administration, advantageous for convenience and potential outpatient therapy. It shows activity against *Candida auris* and *Pneumocystis* spp. but is only available intravenously, as attempts at subcutaneous formulation have been unsuccessful [86].

Ibrexafungerp (SCY-078) is the first representative of a new class of orally administered triterpenoid antifungals approved by the FDA in June 2021 for the treatment of acute and recurrent vulvovaginal candidiasis. It inhibits β-(1,3)-D-glucan synthase, like echinocandins, but by binding to a different site on the enzyme, giving it activity even against some echinocandin-resistant strains [87]. Advantages include oral formulation, activity against a broad spectrum of *Candida species* (including *C. auris* and azole/echinocandin-resistant strains) and *Aspergillus* spp., anti-biofilm properties, good tissue penetration, and limited drug interactions [88]. It is being studied for invasive candidiasis [89], but is not active against *Mucorales* and *Fusarium*.

Olorofim (F901318) is the progenitor of a new class of antifungals, the orotomides, which can be administered orally. It acts by inhibiting dihydroorotate dehydrogenase (DHODH), a key enzyme in the biosynthetic pathway of pyrimidines, which are essential for the synthesis of DNA, RNA, and cell wall precursors. This mechanism is new in the antifungal field. It is active against many difficult-to-treat molds, including azole-resistant strains of *Aspergillus*, *Lomentospora* (formerly *Scedosporium*), *Fusarium*, and endemic dimorphic fungi such as *Coccidioides* [90]. Advantages lie in the oral route of administration, activity against pathogens with limited therapeutic options, and anti-biofilm activity. However, it is not active against yeasts (*Candida*, *Cryptococcus*) or *Mucorales*, and has potential CYP450-mediated drug interactions. It is currently in Phase 3 clinical trials (e.g., OASIS study for invasive aspergillosis) and has received special designations from the FDA (Breakthrough Therapy, Orphan Drug) and EMA (Orphan Drug), but has not yet been approved for marketing [91].

Fosmanogepix (APX001), a propharmaceutical of manogepix, is the first inhibitor of the fungal enzyme Gwt1, which is essential for the maturation of proteins anchored by glycosylphosphatidylinositol (GPI). It thus acts on a novel target, disrupting the proper anchoring of key proteins to the fungal membrane and cell wall. It possesses a broad spectrum of action including *Candida* (also *C. auris* and resistant strains), *Aspergillus*, *Cryptococcus*, *Fusarium*, *Scedosporium*, endemic fungi and some *Mucorales (Rhizopus)*. It is available in both intravenous and oral formulation. Advantages include novel mechanism, broad spectrum and favorable safety profile in preliminary studies [92]. It is not active against *C. krusei* [93]. It has completed Phase 2 studies for candidiasis (including *C. auris*) and is being evaluated for aspergillosis and other rare molds; Phase 3 studies are planned [67].

Oteseconazole (VT-1161) is an oral antifungal agent belonging to the tetrazole class, approved by the FDA in 2022 under the trade name Vivjoa for the treatment of recurrent vulvovaginal candidiasis (RVVC). It acts as a highly selective inhibitor of fungal CYP51, a key enzyme in the biosynthesis of ergosterol. Its high selectivity reduces the risk of drug interactions and side effects compared with conventional triazoles [94]. Oteseconazole has shown activity against fluconazole-resistant strains of *Candida glabrata* and *Candida krusei* due to its unique chemical structure that allows it to bind differently to CYP51 than other azoles [95]. However, its long half-life carries a potential risk of embryo-fetal toxicity, which is why it is not recommended during pregnancy or lactation. The application to the EMA for approval has been withdrawn. Opelconazole (PC945) is a novel triazole formulated for inhaled administration designed to achieve high pulmonary concentrations. It inhibits fungal CYP51, which is essential for ergosterol synthesis, by impairing fungal cell membrane. Inhalation administration achieves high local concentrations in the lung with minimal systemic absorption, thus reducing the toxicity and drug interactions typical of systemically administered azoles. Preclinical and clinical studies have shown that PC945 is well tolerated, with mild side effects and no significant changes in lung function. It is being developed for the treatment and prophylaxis of pulmonary aspergillosis, with particular interest in immunocompromised patients or patients with chronic lung disease [96].

Combining antifungal drugs with different mechanisms of action may improve efficacy, broaden the spectrum of activity, and reduce the risk of resistance development. Combining antifungals with immunotherapies could enhance the host immune response to fungal infections [97]. Combination therapy holds promise for overcoming the limitations of monotherapy, particularly in severe or refractory IFIs and in the context of emerging resistance. Preclinical studies have explored the synergistic effects of antifungal combinations such as azoles + echinocandins. Clinical trials are investigating the use of combination therapies for various IFIs. Immunotherapies such as IFN-γ and monoclonal antibodies are being studied in combination with antifungals [98]. Humanization of monoclonal antibodies for the treatment of mucormycosis is an area of active research [98]. The monoclonal antibody VX-01 targets mucormycosis by blocking angioinvasion [98]. Although preclinical data on synergistic antifungal combinations are encouraging, stronger evidence from clinical trials is needed to guide widespread adoption of these strategies, especially in LMICs where access to multiple antifungal agents may be limited. The development of targeted immunotherapies offers a new approach to the treatment of IFIs.

## 6. Discussion

IFIs represent a critical but often overlooked health challenge in LMICs, where structural limitations in diagnostics and treatment result in high morbidity and mortality rates [99]. A small number of fungal species-about thirty-account for the vast majority of cases, disproportionately affecting immunocompromised individuals [98]. In Honduras, for example, *Candida* spp. and *Aspergillus* spp. have emerged as major pathogens, underscoring regional patterns that may go undetected without adequate laboratory infrastructure [99]. In patients with diseases such as acute lymphoblastic leukemia or lymphoma, fungal infections often accompany bacterial infections, exacerbating the risk of fatal outcomes [100]. These multifactorial scenarios highlight the urgent need for integrated diagnostic and therapeutic protocols suitable for resource-limited settings.

Access to effective antifungal therapy remains inconsistent in low-income countries. Life-saving drugs such as amphotericin B, fluconazole, flucytosine, and itraconazole are often unavailable or inaccessible [101]. Price disparities and supply shortages limit physicians’ ability to provide timely treatment, leading to treatment delays and increased use of suboptimal alternatives [98]. These constraints are further exacerbated by limited training of health care providers, gaps in fungal diagnostics, and the near absence of local surveillance systems that monitor antifungal resistance and utilization patterns [102,103]. The lack of advanced diagnostic tools, such as antigen-based tests and molecular tests, makes early diagnosis of IFI in district-level hospitals particularly difficult. A comparative overview of estimated mortality rates and availability of antifungals in different settings is given in Table 5.

Epidemiological data on IFIs in low-income countries are scarce, making it difficult to quantify their burden or develop evidence-based interventions. The failure to report high-risk pathogens such as *Candida auris*, despite its resistance profile and lethality, reflects the extent of this information gap [34]. It is likely that many infections go undiagnosed and potential outbreaks go unnoticed. This invisibility perpetuates inaction, while the real toll of IFIs—compounded by comorbidities such as HIV and tuberculosis—continues to grow.

The economic toll is equally significant. Treatment of IFIs often involves prolonged hospital stays and costly antifungal regimens, putting a strain on health systems already struggling [104]. In countries with limited health budgets, high drug prices often lead to the use of older and more toxic drugs, which can contribute to worse outcomes for patients [105]. These conditions perpetuate a cycle in which lack of access, delays in treatment, and poor outcomes reinforce each other, exacerbating existing health inequalities on a global scale.

Current conventional culture-based diagnostic tests for IFIs are slow and often characterized by poor sensitivity [106]. At present, blood tests identify only about 40% of life-threatening *Candida* infections. Microscopy requires considerable expertise, and cultures can take several days to provide results. In LMICs, antifungal susceptibility testing is rarely performed [99]. The limitations of current diagnostic methods lead to delays in diagnosis, inappropriate treatment, and increased mortality, especially in LMICs where access to advanced laboratories is limited. New diagnostic technologies such as β-D-glucan assays, PCR-based tests, and metagenomics offer the potential for earlier and more accurate diagnosis. Antigen-detection tests are available but not widely accessible in LMICs. Molecular tests are often absent altogether in many LMICs. These emerging technologies hold promise for overcoming the limitations of traditional diagnostics, but their implementation in LMICs faces challenges related to cost, infrastructure, and trained personnel. Point-of-care tests (POCTs) are needed in LMICs for faster, accurate, inexpensive, and easier testing for priority fungal pathogens [103]. GAFFI aims to implement POCTs for Pneumocystis pneumonia and disseminated histoplasmosis in LMICs [107]. Rapid screening for cryptococcal antigen (CrAg) for people living with HIV/AIDS is available and recommended [108]. Implementation research is needed to understand optimal ways to introduce combined diagnostics and treatments in different settings [107]. POCTs have the potential to revolutionize the management of IFIs in LMICs by enabling timely diagnosis and initiation of treatment at the community level, reducing delays and improving patient outcomes. Table 6 summarizes the main advantages, limitations, and current availability in LMICs of both conventional and emerging diagnostic methods for IFIs. Among nonculture-based diagnostic methods, serum tests for (1→3)-β-D-glucan (BDG) and galactomannan (GM) are widely used biomarkers. BDG serves as a panfungal marker, helping in the diagnosis of candidiasis and aspergillosis; however, it typically gives negative results in infections caused by *Cryptococcus* and Mucorales [109]. Similarly, GM is valuable for the early diagnosis of invasive aspergillosis, particularly when measured in serum and bronchoalveolar lavage (BAL) samples, but has no diagnostic utility for *Candida*, *Cryptococcus*, or Mucorales infections. Both tests are susceptible to false-positive results due to factors such as administration of certain antibiotics or intravenous products [110,111]. In addition, their availability remains limited in many low-income countries [112].

There are currently no antifungal vaccines approved for widespread clinical use. Vaccination could offer a transformative approach to IFI prevention, especially among immunocompromised individuals and in regions with limited access to antifungal therapies. One promising candidate is NDV-3A [113], a recombinant vaccine targeting the adhesin/invasin protein Als3p. In a Phase 2, randomized, double-blind, placebo-controlled study involving women with recurrent vulvovaginal candidiasis (RVVC), a single intramuscular dose of NDV-3A was safe and elicited a robust B- and T-cell response, significantly reducing symptom recurrences over 12 months, with a more pronounced benefit in women younger than 40 years of age. NDV-3A also demonstrated cross-protection against Candida auris: in mouse models, vaccination induced humoral and cellular immunity, leading to improved survival and reduced organ fungal load after challenge with *Candida auris* [114].

Other vaccine candidates currently under investigation include conjugate vaccines directed against *Cryptococcus neoformans*, such as glucuronoxylomannan-tetanus toxoid (GXM-TT), which elicited protective antibody responses and increased survival in mouse models, with early phase clinical trials underway in both healthy and HIV-infected volunteers [115]. For Aspergillus fumigatus, recombinant protein vaccines, such as those based on Asp f3, have shown promise in preclinical studies. In immunosuppressed mice, vaccination with rAsp f3 conferred CD4+ T-cell-mediated protection against invasive aspergillosis.

The advent of mRNA vaccine platforms, validated during the COVID-19 pandemic, has potential for antifungal vaccines due to rapid development and scalability. Success will depend on identifying immunogenic fungal antigens that elicit sustained protection without adverse inflammation.

However, the development of effective antifungal vaccines faces unique obstacles. Fungal pathogens are eukaryotic and are able to evade the immune system through mechanisms such as masking of pathogen-associated molecular patterns, modulation of dendritic cells, and suppression of Th1/Th17 responses. In addition, the primary target populations-patients with hematologic malignancies, transplant recipients, and advanced HIV-are often immunocompromised, which complicates immune responsiveness and clinical trial design [116]. Overcoming these challenges will require new adjuvants or delivery systems, robust antigen discovery pipelines, and adaptive clinical trial strategies tailored to immunodeficient hosts.

To address the burden of IFIs, a multifaceted strategy is needed, as summarized in Table 7, which includes: (i) continued support for antifungal research and development, including public-private partnerships and incentives for pharmaceutical innovation; (ii) expansion of clinical mycology expertise and laboratory capacity, particularly in LMICs; (iii) establishment of real-time global surveillance systems for antifungal resistance; (iv) the integration of fungal infections into national and international health policy frameworks, including pandemic preparedness plans; and (v) the development and implementation of antifungal stewardship programs (ASPs) to optimize treatment decisions and preserve the efficacy of existing drugs.

Systematic reviews and meta-analyses have consistently shown that ASPs significantly reduce antifungal consumption—by 12–59%—and decrease inappropriate antifungal prescriptions without negatively impacting clinical outcomes [80,117]. Core components of effective ASPs include timely initiation of appropriate antifungal therapy, diagnostic stewardship (e.g., targeted use of fungal biomarkers or early microbiologic testing), dose optimization, therapeutic drug monitoring (especially for azoles), de-escalation when possible, and interdisciplinary collaboration involving infectious disease specialists, microbiologists, pharmacists, and intensivists.

Institutional studies corroborate these outcomes: for example, a non-compulsory antifungal stewardship program implemented in a Greek tertiary-care hospital achieved a 42% reduction in antifungal consumption and a 27% decrease in pharmacy acquisition costs, although in-hospital mortality and length of stay remained unchanged [118]. Similarly, implementation of an ASP at the All India Institute of Medical Sciences (AIIMS), New Delhi, increased appropriate antifungal use from 72.6% to 77.9% and reduced untreated IFI cases from 25% to 18.9% [119]. A UK tertiary center reported a 25% reduction in antifungal consumption and a 60% reduction in antifungal expenditure following implementation of a multidisciplinary ASP, with an impressive 93% acceptance rate of stewardship recommendations [120]. These examples highlight the tangible clinical and economic benefits of ASPs across diverse healthcare systems.

However, implementation in LMICs remains particularly challenging. Barriers include limited access to rapid and accurate diagnostics, inadequate laboratory capacity, absence of local guidelines, and lack of dedicated infectious disease specialists or clinical pharmacists. Inconsistent availability of antifungal agents, coupled with budget constraints and fragmented health infrastructure, further complicates stewardship efforts. Moreover, data on local epidemiology and resistance patterns are often scarce, impeding evidence-based decision-making. In such settings, ASPs should be pragmatically adapted to local resource levels, with emphasis on point-of-care diagnostic training, simplified treatment algorithms, and integration with broader AMR and infection prevention and control (IPC) frameworks [102,121]. International collaboration, capacity building, and investment in surveillance systems will be essential to scale up effective antifungal stewardship globally.

## 7. Conclusions

IFIs remain one of the most under-recognized yet consequential global health threats. Affecting millions each year and associated with high mortality, especially in immunocompromised populations and low-resource settings, IFIs continue to receive insufficient attention in global health policy, funding, and research agendas. This neglect persists despite the rising burden of antifungal resistance, the emergence of multidrug-resistant pathogens such as *Candida auris*, and increasing environmental pressures that exacerbate transmission and therapeutic failure.

Over the past decade, important progress has been made in antifungal drug development. Novel agents—such as ibrexafungerp, rezafungin, olorofim, and fosmanogepix—have shown promise in overcoming longstanding pharmacological limitations, including poor oral bioavailability, toxicity, and narrow spectrum. Likewise, technological advances in diagnostics, including molecular assays and point-of-care testing, offer new opportunities for earlier detection and more precise therapeutic targeting. Nevertheless, these innovations remain largely inaccessible in many LMICs, where the burden of IFIs is often highest. The limited availability of essential antifungal drugs, lack of diagnostic infrastructure, and shortage of trained personnel significantly impair the quality of care and widen global inequities.

To close these gaps, action is needed across multiple domains. Investment in antifungal research and development must increase, supported by incentives for public–private collaboration. Initiatives such as the UK-funded The Fungal AMR Innovations for LMICS: Solutions and Access For Everyone (FAILSAFE) project and training programs in global research sponsored by National Institute of Allergy and Infectious Diseases (NIAID) and Fogarty International Center (FIC) [122,123] are essential, but not sufficient on their own. A broader investment by governments, international organizations, and philanthropic foundations is urgently needed to advance understanding of IFIs and develop effective countermeasures. Integrating fungal infections into pandemic preparedness frameworks will ensure a more comprehensive and resilient global health security architecture. Diagnostic and laboratory capacity should be expanded—particularly in LMICs—alongside the training of clinical mycology specialists. Surveillance of antifungal resistance must become more systematic and globally integrated. Most importantly, access to essential antifungal medicines must be secured and stewardship programs implemented widely to ensure appropriate use, reduce toxicity, and limit the development of resistance.

Climate change, agricultural fungicide use, and increasing numbers of immunocompromised patients have created a dynamic threat landscape. The rise of azole resistance in *Aspergillus fumigatus*, partly driven by environmental exposures, underscores the need for a holistic “One Health” approach that incorporates human, environmental, and agricultural dimensions. Likewise, neglected endemic mycoses such as cryptococcal meningitis and histoplasmosis continue to claim lives in regions where diagnostic tools and treatment options are insufficient.

Fungal diseases must be recognized as a priority within national and international infectious disease frameworks, including pandemic preparedness plans. The integration of IFI-specific targets into AMR strategies, financing mechanisms, and global health security agendas is long overdue. Failure to act decisively now risks a future in which currently manageable fungal infections become untreatable.

Ultimately, reducing the burden of IFIs will require not only scientific innovation but also political will, multisectoral coordination, and global equity in implementation. The knowledge, tools, and frameworks exist; what is urgently needed is their activation at scale.

## Figures and Tables

**Table 1:** Summary of the main classes of systemic antifungals. Abbreviations: AUC = Area Under the Concentration-Time Curve, a pharmacokinetic measure of total drug exposure over time; MIC = Minimum Inhibitory Concentration, the lowest drug concentration that inhibits visible fungal growth; Cmax = Maximum Serum Concentration, the peak level of drug in the bloodstream; T>MIC = Time above the MIC, indicating the duration the drug concentration remains above the MIC (important for time-dependent drugs); 5-FU = 5-Fluorouracil, a cytotoxic metabolite derived from flucytosine that disrupts fungal DNA/RNA synthesis; CYP = Cytochrome P450, a family of enzymes involved in drug metabolism and interactions; ABC/MFS = ATP-Binding Cassette/Major Facilitator Superfamily, efflux pumps contributing to antifungal resistance by expelling drugs from the fungal cell.

Antifungal Class	Specific Molecular Target	Main Mechanism of Action	Activity vs. Key Pathogens	Primary PK/ PD Index	Main Known Resistance Mechanisms	Notes
Azoles (Triazoles: Fluconazole, Itraconazole, Voriconazole, Posaconazole, Isavuconazole)	Lanosterol 14α-demethylase (Erg11/Cyp51A)	Inhibits ergosterol synthesis, accumulation of toxic methylated sterols	Fungistatic (*Candida*, *Aspergillus*, *Cryptococcus*, dimorphic fungi—variable spectrum)	AUC/MIC [23]	*ERG11*/*cyp51A* mutations/ overexpression, efflux pump overexpression (ABC/MFS) [27]	Significant CYP P450 interactions, Hepatotoxicity [23]
Polyenes (Amphotericin B)	Membrane ergosterol	Binds ergosterol, pore/channel formation, alters membrane permeability	Fungicidal (Broad spectrum: *Candida*, *Aspergillus*, *Cryptococcus*, *Mucorales*, dimorphic fungi)	Cmax/MIC [23]	Decreased ergosterol content, membrane alterations (rare); intrinsic resistance (e.g., *A. terreus*) [28]	Nephrotoxicity, infusion reactions (reduced with lipid formulations) [23]
Echinocandins (Caspofungin, Micafungin, Anidulafungin, Rezafungin)	β-(1,3)-D-glucan synthase (Fks1/Fks2)	Inhibits β-(1,3)-D-glucan synthesis, damages cell wall	Fungicidal (*Candida* spp.), Fungistatic (*Aspergillus* spp.)	AUC/MIC, Cmax/MIC [24]	Hotspot mutations in *FKS1*/*FKS2* genes [29]	Well tolerated, low CYP interaction potential, IV only, limited spectrum (inactive vs *Cryptococcus*, *Mucorales*) [23]
Allylamines (Terbinafine)	Squalene epoxidase (Erg1)	Inhibits ergosterol synthesis, accumulation of toxic squalene	Fungicidal (dermatophytes)	Not well defined for systemic IFI	*ERG1*/*SQLE* mutations/ overexpression [30]	Limited systemic use (dermatomycoses), good tolerability [23]
Flucytosine (5-FC)	DNA and RNA synthesis	Intracellular conversion to 5-FU, inhibits thymidylate synthase and incorporates into RNA	Fungistatic (*Candida* spp., *Cryptococcus* spp.)	T > MIC	Mutations in permeases or metabolic enzymes (cytosine deaminase, UMP pyrophosphorylase) [31]	Almost always used in combination, Myelotoxicity [24]

**Table 2:** Geographic distribution of antifungal resistance rates by pathogen and drug. Values are based on the most recent published surveillance data. When available, specific country data and prevalence ranges are provided. “TR%” refers to environmentally derived azole resistance associated with mutations such as TR34/L98H and TR46/Y121F/T289A.

Pathogen-Drug Combination	North America	South America	Europe	Asia	Africa	Middle East	Oceania	Notes/Key Sources
*A. fumigatus*—Azoles (Clinical)	<5%	>10% in Brazil	>10–20% (NL, UK, Spain); <10% elsewhere	10–20% (China, Japan)	No robust data	>15% in Iran	<5% (sparse data)	Linked to environmental TR%; [28,59]
*A. fumigatus*—Azoles (Environmental, TR%)	<5%	5–15% (patchy)	10–20% (NL, UK, Spain)	30–80% (China, Vietnam)	5–10% (few studies)	>50% (Iran)	5–10% (limited)	Main markers: TR34/L98H, TR46/Y121F/ T289A [55]
*C. auris*—Fluconazole	>90%	>90%	>90%	>90%	91% (South Africa)	>90%	>90%	Near-universal resistance [32,34]
*C. auris*—Amphotericin B	8–15%	33% (Colombia)	10–30% (varies by country)	10–30% (India)	21% (South Africa)	10–20% (limited)	10–20% (limited)	Resistance varies by clade/region [61,62]
*C. auris*—Echinocandins	<5%, but outbreaks reported	<2%	<5%, occasional outbreaks	<5%	1.7% (South Africa)	<5%	<5%	Generally susceptible; resistance emerging [34,35]
*C. glabrata*—Fluconazole	15–20%	Insufficient data	86–100% (Slovenia, Croatia)	Patchy, scarce data	No reliable data	No reliable data	Insufficient data	High resistance in some regions [45,63]
*C. glabrata*—Echinocandins	10–15% (USA)	Insufficient data	<5%	<5%	No reliable data	No reliable data	No reliable data	Emerging resistance in USA [61]
*C. parapsilosis*—Fluconazole	<10%	15% (pooled)	73–81% (Italy, Croatia)	15% (pooled)	Sparse data	Sparse data	Sparse data	High resistance in selected countries [45,50]

**Table 3:** Summary of key invasive fungal infections, recommended first-line treatments, and alternative (backup) therapies. Choice may vary based on patient condition, local resistance, and drug availability.

Infection/Pathogen	First-Line Therapy	Alternative (Backup) Therapy
*Cryptococcal meningitis* (*Cryptococcus neoformans*)	Amphotericin B (lipid or deoxycholate) + Flucytosine (induction), followed by Fluconazole (maintenance)	Fluconazole monotherapy (if flucytosine unavailable), or Amphotericin B alone
*Invasive candidiasis* (*Candida* spp.)	Echinocandin (e.g., caspofungin, micafungin)	Fluconazole (in stable patients), Liposomal Amphotericin B (if echinocandins unavailable)
*Invasive aspergillosis* (*Aspergillus fumigatus*)	Voriconazole or Isavuconazole	Liposomal Amphotericin B; Posaconazole as salvage therapy
*Mucormycosis* (*Rhizopus*, *Mucor*)	Liposomal Amphotericin B	Posaconazole or Isavuconazole
*Pneumocystis jirovecii* pneumonia (PJP)	Trimethoprim–sulfamethoxazole (TMP–SMX)	Pentamidine, Atovaquone, or Dapsone + Trimethoprim
*Disseminated histoplasmosis* (*Histoplasma capsulatum*)	Liposomal Amphotericin B (initial), followed by Itraconazole	Amphotericin B deoxycholate (if lipid formulation unavailable), Fluconazole (less effective)

**Table 4:** Novel antifungal agents approved or in advanced development. Structural descriptions summarize the representative chemical scaffold of each compound or class. PO = oral, IV = intravenous, Inh. = inhaled, RVVC = recurrent vulvovaginal candidiasis.

Drug Name	Class/ Mechanism	Primary Target Pathogens	Route(s)	Key Advantage(s)	Limitation(s)	Development Status	Representative Chemical Structure
*Rezafungin*	Echinocandin (Glucan synthase inhibitor)	*Candida* spp. (incl. *C. auris*)	IV	Weekly administration (long half-life)	IV-only, spectrum similar to other echinocandins	Approved (Candidemia)	Cyclic lipopeptide with hexapeptide core and lipid side chain
*Ibrexafungerp*	Triterpenoid (Glucan synthase inhibitor—distinct Fks site)	*Candida* spp. (incl. resistant), *Aspergillus* spp.	PO	Oral, activity against echinocandin-resistant strains	Inactive against *Mucorales*/*Fusarium*	Approved (RVVC)	Semi-synthetic triterpenoid (enfumafungin derivative) with polycyclic core
*Olorofim*	Orotomide (DHODH inhibitor – pyrimidine synthesis)	Molds (e.g., resistant *Aspergillus*, *Lomentospora*, *Scedosporium*)	PO	Novel mechanism, oral, active against difficult molds	Inactive against yeasts/*Mucorales*, potential CYP450 interactions	Phase 3	Diarylamide core with substituted pyrimidine ring
*Fosmanogepix*	Gwt1 inhibitor (GPI-anchored protein maturation)	Broad spectrum (*Candida*, *Aspergillus*, *Cryptococcus*, rare molds)	IV, PO	Novel mechanism, broad spectrum, IV/PO options	Inactive against *C. krusei*	Phase 2/3 planned	Prodrug of manogepix: hydroxypyridazinone core with phosphonooxymethyl group
*Oteseconazole*	Tetrazole (Selective CYP51 inhibitor)	*Candida* spp. (incl. fluconazole-resistant)	PO	Oral, high CYP51 selectivity (potentially fewer interactions/toxicity)	Potential embryofetal risk, long half-life, only for recurrent VVC	Approved (RVVC, USA)	Tetrazole ring replacing triazole of azoles, with substituted aromatic moieties
*Opelconazole*	Triazole (CYP51 inhibitor)	*Aspergillus* spp.	Inh.	High lung concentrations, low systemic exposure	Pulmonary-only, likely needs adjunct systemic therapy	Phase 2/3	Triazole core structure with fluorinated phenyl side chains

**Table 5:** Estimated mortality rates for selected invasive fungal infections and availability of key antifungal agents in HICs vs. LMICs. Mortality rates in LMICs are often approximated due to limited data.

Invasive Fungal Infection	Estimated Mortality Rate (%) in High-Income Countries (HICs)	Estimated Mortality Rate (%) in LMICs
*Cryptococcal meningitis*	9–15	22–96
*Invasive aspergillosis*	30–50	70–90 (est.)
*Invasive candidiasis*	20–40	40–70 (est.)
*Pneumocystis jirovecii* pneumonia	10–20	30–50 (est.)
**Antifungal Agent**	**Availability in HICs**	**Availability in LMICs**
Fluconazole	Widely available	Limited
Amphotericin B (deoxycholate/lipid)	Widely available	Limited
Flucytosine	Available	Rarely available or absent
Voriconazole	Available	Limited
Echinocandins	Available	Limited

**Table 6:** Comparison of current and emerging diagnostic methods for invasive fungal infections.

Diagnostic Method	Key Advantages	Key Limitations (Including LMIC Contexts)	Current Availability in LMICs
Culture	Standard method for identification; allows antifungal susceptibility testing	Slow (days to weeks); variable sensitivity; may be difficult to distinguish colonization from infection	High
Microscopy	Rapid; inexpensive	Requires expertise; variable sensitivity; not always specific	High
β-D-Glucan Test	Pan-fungal marker; useful for early detection of candidiasis and aspergillosis	Non-specific; false positives (e.g., hemodialysis, IVIG); not useful for Mucorales or Cryptococcus	Low
Galactomannan (GM) Test	Useful for early diagnosis of invasive aspergillosis; can be performed on serum or BAL	Specific for Aspergillus; false positives (e.g., antibiotics, foods); not useful for Candida, Cryptococcus, or Mucorales	Low
PCR-based Tests	Rapid; high sensitivity and specificity	Requires specialized equipment and expertise; costly; may not distinguish viable organisms from DNA; limited availability in LMICs	Low
Antigen Detection	Rapid; may be point-of-care (e.g., CrAg for Cryptococcus)	Variable specificity; limited availability for many pathogens in LMICs	Low
Metagenomics	Detects broad range of organisms; may identify rare or mixed infections	Expensive; requires bioinformatics expertise; complex interpretation; extremely limited availability in LMICs	Very Low
Lateral Flow Assays (LFA)	Rapid; point-of-care; potentially inexpensive	Variable sensitivity and specificity depending on test; limited availability for many pathogens	Low

**Table 7:** Strategic areas and recommendations to strengthen the global response to IFIs.

Action Area	Specific Recommendations	Stakeholders
Policy and Awareness	Officially recognize IFIs as a global health priority; integrate IFIs into pandemic preparedness plans	WHO, Governments, Regulatory Bodies
Research and Development	Increase funding for basic research, development of new antifungals, diagnostics, and vaccines; promote translational and clinical research	Governments, Funding Agencies, Pharmaceutical Industry, Research Institutions
Diagnostics	Invest in the development and distribution of rapid, accurate, and affordable diagnostic tests, including POCTs, especially in LMICs; strengthen laboratory capacity in LMICs	WHO, Governments, Funding Agencies, Diagnostic Industry
Treatment and Antifungal Stewardship	Ensure access to essential and affordable antifungal drugs in LMICs; implement and strengthen antifungal stewardship programs in clinical and hospital settings; develop treatment guidelines tailored to LMICs	WHO, Governments, Healthcare Providers, Pharmaceutical Industry
Capacity Building	Train healthcare workers in LMICs in IFI diagnosis, management, and prevention; develop mycology reference centers and expert networks	WHO, Governments, Academic Institutions, Non-Governmental Organizations
Financing	Increase public and philanthropic funding for research, development, and implementation of IFI-related interventions	Governments, Funding Agencies, Philanthropic Foundations
